# Actively transcribed and expressed *atp8* gene in *Mytilus edulis* mussels

**DOI:** 10.7717/peerj.4897

**Published:** 2018-06-08

**Authors:** Marek Lubośny, Aleksandra Przyłucka, Beata Śmietanka, Sophie Breton, Artur Burzyński

**Affiliations:** 1 Department of Genetics and Marine Biotechnology, Institute of Oceanology Polish Academy of Sciences, Sopot, Poland; 2 Department of Biological Sciences, Université de Montréal, Montréal, Québec, Canada

**Keywords:** ATP8, Western blot, Bivalvia, Blue Native, Mitochondrial DNA, Doubly uniparental inheritance

## Abstract

**Background:**

Animal mitochondrial genomes typically encode 37 genes: 13 proteins, 22 tRNAs and two rRNAs. However, many species represent exceptions to that rule. Bivalvia along with Nematoda and Platyhelminthes are often suspected to fully or partially lack the ATP synthase subunit 8 (*atp8*) gene. This raises the question as to whether they are really lacking this gene or is this maybe an annotation problem? Among bivalves, *Mytilus edulis* has been inferred to lack an ATP8 gene since the characterization of its mitochondrial genome in 1992*.* Even though recent bioinformatic analyses suggested that *atp8* is present in *Mytilus* spp., due to high divergence in predicted amino acid sequences, the existence of a functional *atp8* gene in this group remains controversial.

**Results:**

Here we demonstrate that *M. edulis* mitochondrial open reading frames suggested to be *atp8* (in male and female mtDNAs) are actively translated proteins*.* We also provide evidence that both proteins are an integral part of the ATP synthase complex based on in-gel detection of ATP synthase activity and two-dimensional Blue-Native and SDS polyacrylamide electrophoresis.

**Conclusion:**

Many organisms (e.g., Bivalvia along with Nematoda and Platyhelminthes) are considered to be lacking certain mitochondrial genes often only based on poor similarity between protein coding gene sequences in genetically closed species. In some situations, this may lead to the inference that the ATP8 gene is absent, when it is in fact present, but highly divergent. This shows how important complementary role protein-based approaches, such as those in the present study, can provide to bioinformatic, genomic studies (i.e., ability to confirm the presence of a gene).

## Introduction

The blue mussel *Mytilus edulis* is one of the bivalve species possessing the unusual system of doubly uniparental inheritance (DUI) of mitochondria (Skibinski, Gallagher & Beynon, 1994; Boore, Medina & Rosenberg, 2004). Contrary to strictly maternal inheritance (SMI) of mtDNA found in other animal species, male *Mytilus* spp. mussels have two different mitogenomes. One is inherited from the father (M-type mtDNA; located mainly in male germ line cells), and the second from the mother (*F*-type mtDNA; located in female germ line cells and in somatic cells of both sexes) (Zouros et al., 1994). Estimated K2P genetic distance (Kimura two-parameter corrected for multiple substitutions) between M and F mtDNAs reaches 0.245 (Zbawicka, Burzyński & Wenne, 2007), and while the presence of two mitogenomes (heteroplasmy) has been demonstrated several times using DNA-based methods (Garrido-Ramos et al., 1998; Dalziel & Stewart, 2002; Zbawicka, Skibinski & Wenne, 2003; Obata et al., 2006; Kyriakou, Zouros & Rodakis, 2010), proteomic approaches have not been much explored yet (Chakrabarti et al., 2006). Table 1 shows mean pairwise distance between male and female *M. edulis* mitochondrial proteins, which reaches 0.386 for ATP8.

**Table 1:** Mean pairwise distance between male and female *M. edulis* mitochondrial proteins (GenBank HM489874 and MF407676) (Zbawicka, Burzyński & Wenne, 2007; Kumar, Stecher & Tamura, 2016).

Protein	Mean pairwise distance
COX1	0.047
COX3	0.093
COX2	0.112
ND3	0.112
CYTB	0.140
ND4	0.143
ATP6	0.155
NAD4L	0.172
ND1	0.186
ND5	0.196
ND2	0.210
ND6	0.221
^*^ATP8	0.386

**Notes.**

* The *atp8* gene lacks annotation.

*M. edulis* was the first bivalve species with a nearly completely sequenced mitogenome (Hoffmann, Boore & Brown, 1992). The F-type mtDNA was annotated with 37 genes: two ribosomal RNAs, 23 tRNAs (with an additional *tRNA*^*met*^ gene) and only 12 protein-coding genes, i.e., the mitogenome was lacking the *atp8* gene (GenBank Acc. No. AY484747 (Hoffmann, Boore & Brown, 1992)). This publication created a belief that bivalves might lack *atp8*, although a few bivalvian mitogenomes published later had this gene annotated. Putative *atp8* gene in *Mytilus* F and M mitochondrial genomes was identified in 2010 (Breton, Stewart & Hoeh, 2010; Smietanka, Burzyński & Wenne, 2010). Difficulties with the annotation of this gene most probably originated from the interspecies differences in coded protein amino acid sequence.

**Figure 1:**
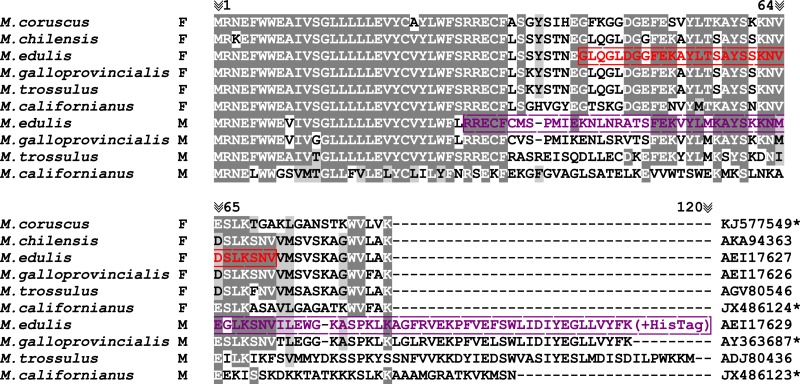
*Mytilus. spp.* ATP8 protein alignment. JX486123; JX486124; KJ577549; AY363687, *Protein sequences extracted from mtDNAs without annotated *atp8* gene. Alignment colored with BoxShade ExPASy online tool with 0.6 shading parameter (grey color). Red color represents FATP8-antigen peptide sequence used for immunisation; violet color represents MATP8-antigen protein sequence used for immunisation (Mizi et al., 2005; Ort & Pogson, 2007; Smietanka, Burzyński & Wenne, 2010; Lee & Lee, 2014; Sańko & Burzyński, 2014; Gaitán-Espitia et al., 2016; Zbawicka, Wenne & Burzyński, 2014).

ATP8 is a part of the non-catalytic hydrophobic membrane component (F_*o*_) of the ATP synthase (F_1_F_*o*_) complex (Rak, Gokova & Tzagoloff, 2011; Lee et al., 2015). It is a short protein composed of 37–70 amino acids (6,784 out of 7,301 ATP8 protein sequences deposited in Genbank RefSeq database), and 94% of known sequences (i.e., 6,873 out of 7,301) start with a conserved MPQ tripeptide and possess one predicted transmembrane domain (Papakonstantinou et al., 1996; Gissi, Iannelli & Pesole, 2008). The predicted ATP8 in *Mytilus* mitochondrial genomes also possesses one predicted transmembrane domain but lacks the MPQ sequence at the N-terminus of the peptide and the length of the protein varies from 84 aa in the F-type mtDNA to 106–128 aa in the M-type mtDNA (Fig. 1).

Bioinformatic analyses of *Mytilus* spp. mitogenomes (Breton, Stewart & Hoeh, 2010; Smietanka, Burzyński & Wenne, 2010) have shown that this “putative *atp8* gene” in both F and M mtDNAs possesses a pattern of evolution expected for a protein-coding gene evolving under purifying selection (i.e., the 3rd>1st>2nd codon pattern of evolution). Furthermore, both F and M sequences are actively transcribed in *Mytilus* species (based on EST sequences), and comparison of protein hydropathy predictions with ATP8 proteins from other bivalves revealed similar profiles (Breton, Stewart & Hoeh, 2010; Smietanka, Burzyński & Wenne, 2010). However, due to its high divergence in predicted amino acid sequences compared to other ATP8, the existence of a functional *atp8* gene in *Mytilus* spp. remains controversial. In fact, some authors have suggested that sequences annotated as *atp8* in *Mytilus* spp. could represent a pseudogene (Uliano-Silva et al., 2016).

Here we demonstrate that the open reading frames (ORFs) suggested to be *atp8* in both *M. edulis* F and M mtDNAs are actively expressed and effectively represent parts of the ATP synthase complex.

## Materials and Methods

### Western blot

Living specimens of *M. edulis* mussels were bought at a local market in January and September 2016 (Collecting area: the Oosterschelde and the Waddenzee bay; Netherlands). The sex of each individual was determined by examination of gonadal tissues for the presence of sperm or eggs under light microscope. Ten pairs of male and female individuals where checked during the course of the experiment. Mantle with gonads, gill, foot and hepatic gland tissues of male and female specimens were carefully sectioned with sterile scalpels to minimize possibility of cross contamination of tissue samples and subsequently washed in sterile water and stored frozen at −20 °C for further analyses. Approximately 100 mg of every tissue type were suspended in 1 ml of Radioimmunoprecipitation assay (RIPA) lysis buffer (50 mM Tris–HCl, 150 mM NaCl, 1 mM ethylenediaminetetraacetic acid, 1% Triton X-100, 0.5% Na-deoxycholate, 0.1% SDS, pH 7,6) supplemented with 5 µl of protease inhibitors cocktail (Sigma-Aldrich, St. Louis, MO, USA), homogenized at 20,000 RPM for 20 s (Heidolph SilentCrusher M; tool 8F; Sigma-Aldrich, St. Louis, MO, USA) and sonicated (Vibra-Cell; Sonics, Newton, CT, USA) for 20 s; amplitude 50%. Samples were kept on ice during the whole protein isolation process. Isolates were then centrifuged at 15,000× g for 4 min, to separate insoluble residues, transferred into new vials and stored frozen or used immediately in further steps. 40–60 µg of crude protein isolates per sample were separated by SDS-PAGE electrophoresis (5% stacking gel; 10% separating gel in 6 M urea to protect low molecular weight bands from diffusing and smearing during electrophoresis; 30 min at 80 V and 1 h at 150 V) with cooling (BlueStar; DNAGdansk; Gdańsk, Poland) and transferred to membrane (OWL electroblotting semidry system). Due to the low molecular weight of targeted proteins (FATP8 9,5∼kDa; MATP8∼13  kDa), 0.2 µm PVDF or nitrocellulose membranes (GE Healthcare, Little Chalfont, UK) were used and electroblotting time did not exceed 30 min at 200 mV (longer transfer times were causing over transfer of small proteins through membranes onto the Whatman filter papers).

The remaining procedures were performed in the standard manner: membrane blocking in 4% low fat dry milk in Phosphate-buffered saline buffer for 1 h, overnight incubation with primary polyclonal antibody dilution 1:5,000 at 4 °C, rinsing with 0.05% Tween-20 in PBS 3 × 5 min, incubation with anti-rabbit secondary HRP-conjugated monoclonal antibody dilution 1:10,000 for 1 h (Sigma-Aldrich, St. Louis, MO, USA), colorimetric immunodetection with DAB (SigmaAldrich) or DAB supplemented with Co or Ni ions. The whole procedure was repeated at least 6 times with different pairs of male and female specimens and all tested specimens gave coherent results.

Polyclonal antibodies were custom-made by GenScript. It is worth mentioning that due to the high similarity of the M and FATP8 protein N-terminus sequences, their low antigenicity and predicted difficulties with solubility, the N-terminal hydrophobic domain of both M and FATP8 proteins has been removed from the sequences at the antibody designing step (Fig. 1). The MATP8 antigen was acquired through expression in bacterial host with HisTag on C-term of the peptide and the FATP8 peptide was chemically synthetized and purified before immunisation. Antibodies were acquired from rabbits after a triple immunization procedure.

#### BN-PAGE/SDS-PAGE

Small specimens of *M. edulis* were sexed and sectioned for mitochondrial isolation (the whole body, around 300 mg, without the hepatic gland was used for the females and the ripe mantle/gonad tissues were used for the male individuals). Tissues were homogenised 10 s at 4,000 RPM (Heidolph SilentCrusher M; tool 8F), with 1.5 ml of 440 mM sucrose, 1 mM ethylenediaminetetraacetic acid, 20 mM 3-(N-morpholino) propanesulfonic acid, 1 mM phenylmethylsulfonyl fluoride, 0.5 mM sodium orthovanadate, pH 7.2 and subsequently centrifugated at 5,000× g for 10 min. The supernatant was discarded and the remaining disrupted tissue pellet was suspended in 1.5 ml isolation buffer (1 M aminocaproic acid, 50 Mm Bis-Tris, 1 mM phenylmethylsulfonyl fluoride, 0.5 mM sodium orthovanadate pH 7.0) and homogenised 10 s at 20,000 RPM (Dabbeni-sala et al., 1997). Whole mitochondria were collected by differential centrifugation with the first centrifugation at 5,000× g for 20 min at 4 °C to remove unbroken cells and bigger cell fragments, followed by a second centrifugation of the remaining supernatant (25,000× g, 20 min, 4 °C) to pellet mitochondria and other small cell organelles. The resultant mitochondrial pellets were then solubilized in 100 µl 19:1 solution of isolation buffer and Triton X-100. After 5 min incubation, samples were centrifuged again at 25,000× g for 10 min at 4 °C. Typical Blue Native 4–15% gradient gel (Eubel, Braun & Millar, 2005; Wittig, Braun & Scha, 2006; Fiala, Schamel & Blumenthal, 2011) was substituted with discontinuous gradient gel (layers of different percentage acrylamide gels 4/5/6/8/10/15%). Before loading, every 15 µl of sample was supplied with 5 µl of 10% Coomassie G-250.

In-gel visualisation of Complex V (ATP synthase) was performed through overnight incubation of Blue Native gel in 35 mM Tris Base, 276 mM Glycine, 14 mM MgCl_2_, 0.2% Pb(NO_3_)_2_ and 8 mM ATP pH∼7.8 as in (Dabbeni-sala et al., 1997). Gel fragments containing stained ATPase complex were then cut out, incubated 45min in SDS-PAGE running buffer to dissociate and denature ATP synthase complexes to individual subunits and loaded horizontally on standard 10% (5% stacking) SDS-PAGE gel. Electrophoresis and western blot procedure were performed as described above. Enzymatic detection was completed according to manufacturer’s protocol with Ultra-Sensitive ABC Peroxidase Rabbit IgG Staining Kit (Thermo Fisher, Waltham, MA, USA). Remaining Blue Native gel fragments were slightly decolorized by longer (1–2 h) incubation in SDS containing Tris-glycine buffer and electroblotted together with gels after two dimensional electrophoresis (presence of Coomassie G-250 in Blue Native gels hinders later detection due to the high binding affinity to the hydrophobic blotting membranes). Two-dimensional BN/SDS PAGE procedure was repeated with at least 6 male and 6 female specimens.

## Results

**Figure 2:**
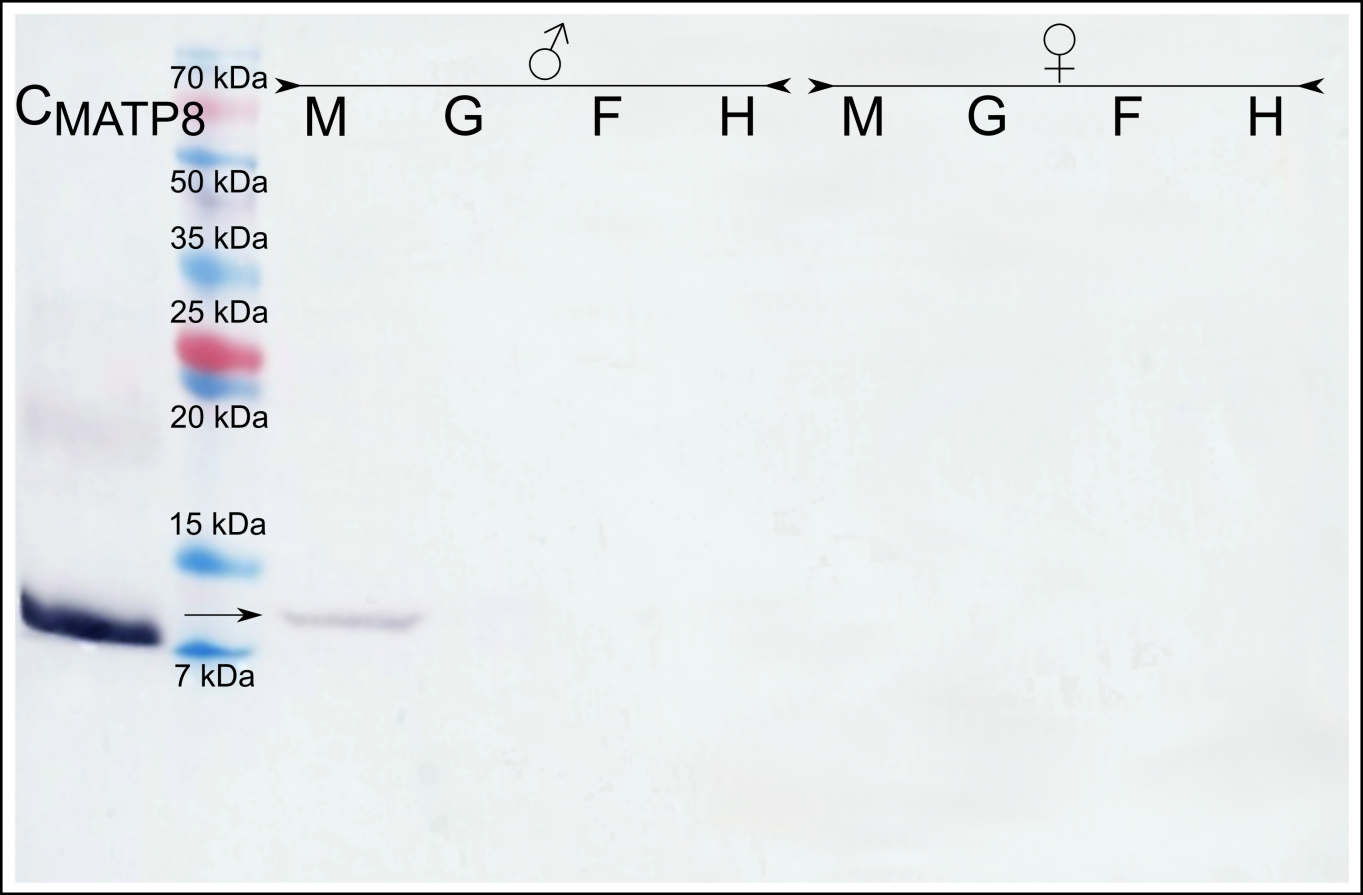
Male ATP8: tissue segregation. C_MATP8_- positive control; M-mantle tissue where gonads are localized in *Mytilus* spp.; G-gills; F-foot; H-hepatic gland, arrow indicates detected male version of ATP8 protein. The signal detected in male mantle/gonad tissue highly corelates with predicted 13.5 kDa molecular weight of MATP8 protein. As expected (Skibinski, Gallagher & Beynon, 1994), MATP8 is absent in all other tissues.

**Figure 3:**
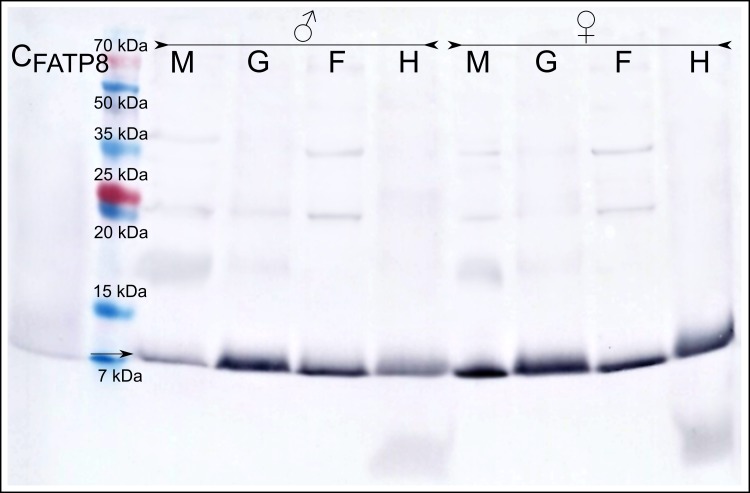
Female ATP8: tissue segregation. C_FATP8_-positive control; M-mantle with gonads; G-gills; F-foot; H-hepatic gland, arrow indicates detected female version of ATP8 protein. Signal for FATP8 (predicted molecular weight 10 kDa) is present in all tissues both in male and female individuals. FATP8 and MATP8 (Fig. 2) are both present in the male mantle tissue containing inseparable gonads and somatic cells.

Immunodetection with the anti-MATP8 antibody gave positive results only in male mantle/gonad tissue (Fig. 2). The size of the signal generated was highly similar to the predicted molecular weight of MATP8 (∼13.5 kDa) and also highly specific with no additional protein bands. No signal was visible in other male and female tissue samples. Contrary to anti-MATP8, anti-FATP8 antibody gave signal for every tissue (mantle, gill, foot, hepatic gland) both for male and female specimens (Fig. 3). This signal also corresponded to the predicted protein molecular weight of FATP8 (∼10 kDa). The signal for FATP8 in male mantle/gonad tissue was visibly weaker than the signals for other tissues. The anti-FATP8 antibody was also less specific then the anti-MATP8. Non-specific bands were detectable on levels corresponding for proteins larger than 15 kDa.

**Figure 4:**
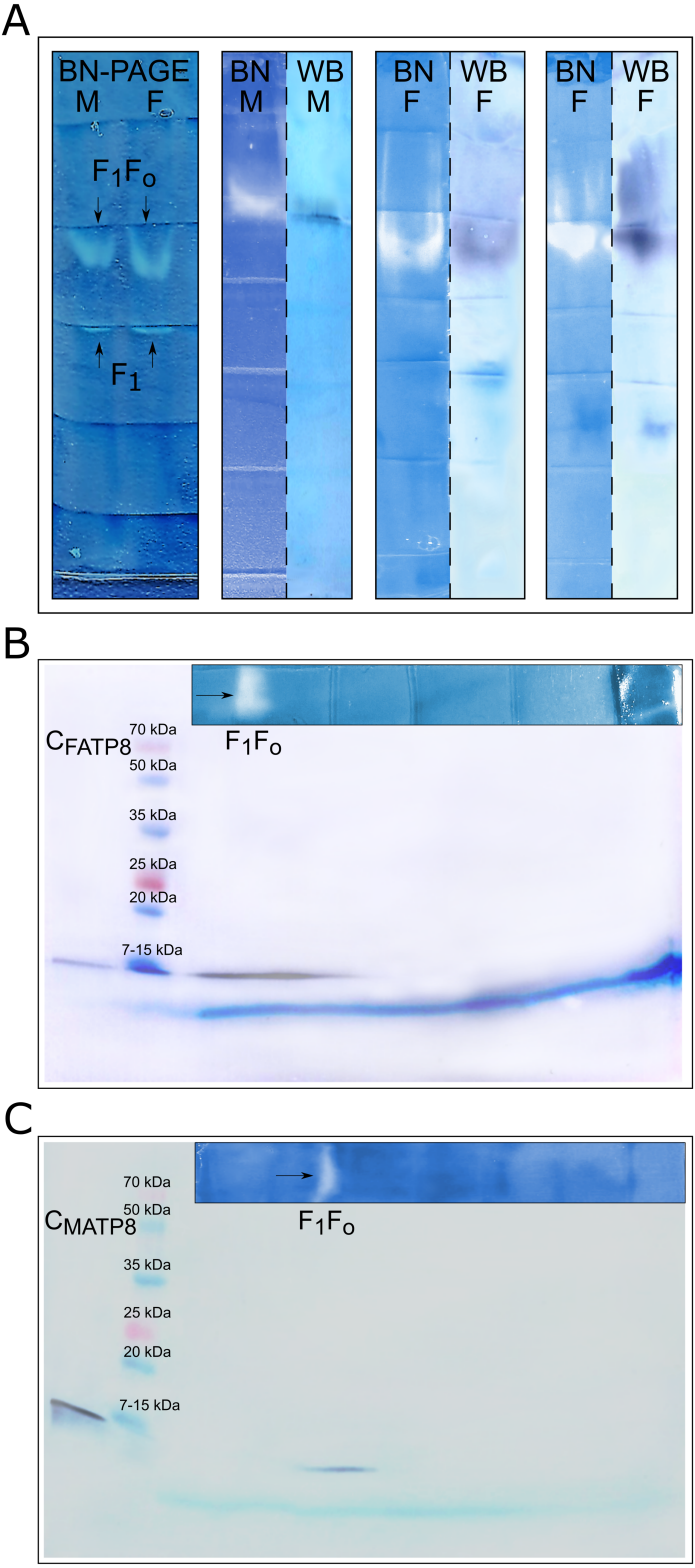
ATP8 as an integral part of the ATPase complex. (A) Enzymatic staining of Blue native gels and immunodetection of F_1_F_*o*_ and F_1_ (catalytic) parts of ATPase complex: white and white-blue bands represent in-gel localization of ATP synthase complex after enzymatic staining; dark purplish blue bands show immunodetection of MATP8 (in M), FATP8 (in F) as well as colocalization of those proteins with ATP synthase complex (white bands in BN); BN-Blue native polyacrylamide gel enzymatic staining, WB-Western blot immunodetection, F-female specimen, M-male specimen. (B) Immunodetection of ATP8 female version after two-dimensional SDS-PAGE electrophoresis: C_FATP8_-control, F_1_F_*o*_-whole ATP synthase complex. (C) Immunodetection of ATP8 male version after two-dimensional SDS-PAGE electrophoresis: C_MATP8_-control, F_1_F_*o*_-whole ATP synthase complex.

In-gel detection (Fig. 4) of F and M ATP synthase activity (after Blue Native electrophoresis) indicated the presence of one or two white lead phosphate precipitates (depending on the sample). The higher band corresponded to the whole Complex V (F_1_F_*o*_), whereas the lower band corresponded to the dissociated catalytic part (F_1_). Blue Native gel parts blotted after slight decolorization from Coomassie G-250 and dissociation in SDS containing Tris-glycine buffer resulted with signals matching in-gel activity spots. Both male and female mitochondrial isolates separated by two-dimensional BN-PAGE/SDS-PAGE electrophoresis also gave positive results. Signals on the membrane were present for separated parts of the whole F and M ATP synthase complexes (F_1_F_*o*_) at expected molecular weight positions. No nonspecific bands were observed.

**Figure 5:**
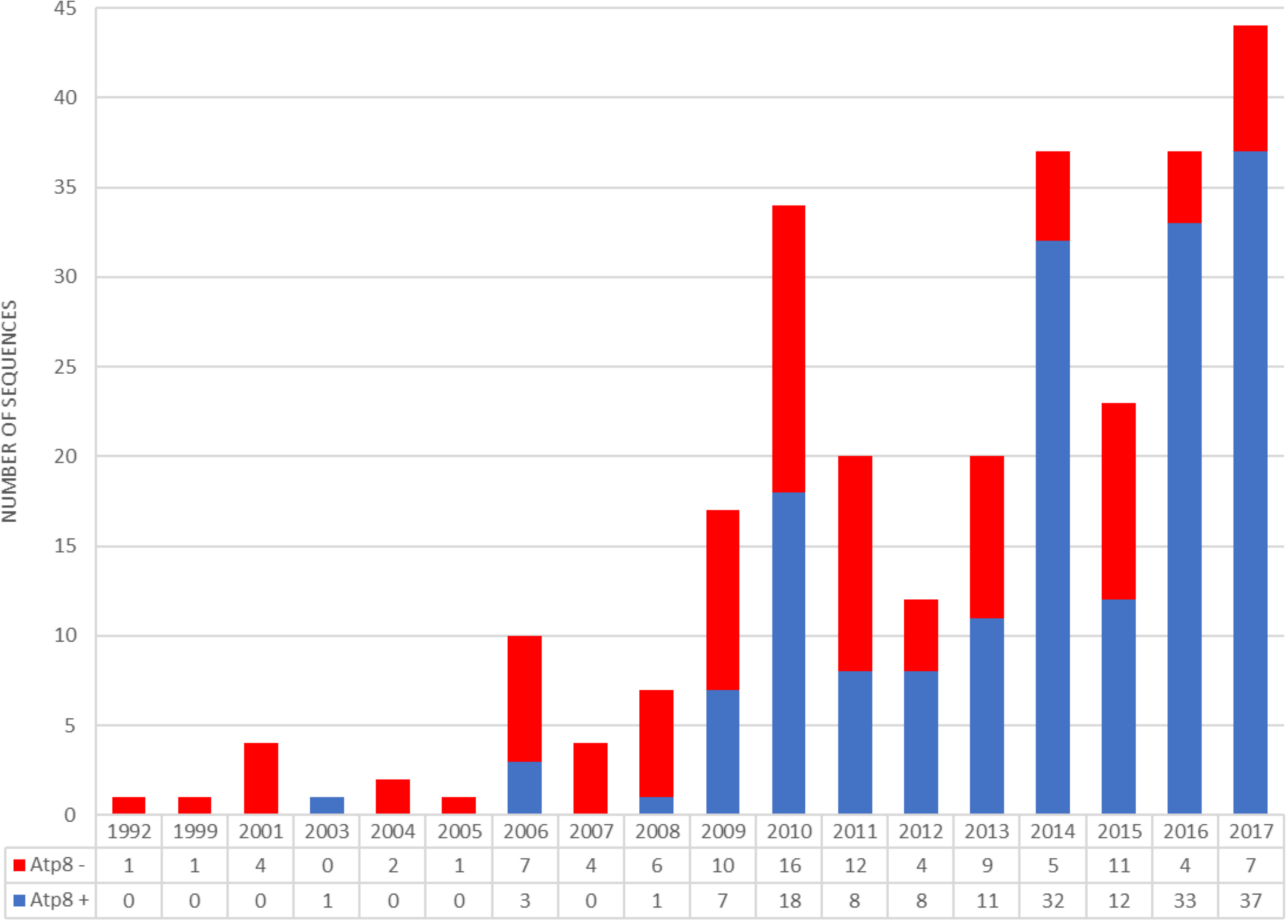
Number of bivalvian mitochondrial genomes with and without annotated *atp8* gene deposited since 1992 in GenBank database.

## Discussion

Before 2009, only five out of 31 bivalve mitochondrial genomes deposited in GenBank have featured *atp8* (Serb & Lydeard, 2003; Dreyer & Steiner, 2006). Since 2009, a constant increase in the number of bivalve mitogenomes annotated with *atp8* has been observed (Fig. 5;  Data S1). However, at the end of 2017 only 62% of all bivalvian mtDNAs available in GenBank had an annotated *atp8* gene and only a small amount (if any at all) of old submissions appeared to have been corrected. Although the percentage of annotated *atp8* in bivalve mitogenomes published between 2010 and 2017 equals 77% ( Data S1), there still remain some scientists publishing bivalve mtDNAs without this gene, even within the genus *Mytilus* (e.g., *M. coruscus* Lee & Lee, 2014), where amino acid sequences for this protein are very similar among species (Fig. 1). As mentioned above, some authors have suggested that sequences annotated as *atp8* in *Mytilus* spp. could represent a pseudogene (Uliano-Silva et al., 2016).

In the present study, protein products coded by both M and F mitochondrial ORFs suggested to be *atp8* were detected in male and female *M. edulis* mussels. The female FATP8 was present in every studied tissue (male and female mantle, gill, foot, hepatic gland). In contrast, MATP8 was shown to be present only in male mantle/gonad, a result that was expected because male mitochondrial genome in *Mytilus* spp*.* has been observed predominantly in gonad tissues and is the only mtDNA present in sperm cells (Skibinski, Gallagher & Beynon, 1994). There are reports suggesting leakage of small amounts of M mtDNA to somatic tissues (Garrido-Ramos et al., 1998; Dalziel & Stewart, 2002; Zbawicka, Skibinski & Wenne, 2003; Obata et al., 2006; Kyriakou, Zouros & Rodakis, 2010). However, the western blot technique is less sensitive than the polymerase chain reaction (PCR), and this could explain why we did not detect the MATP8 in somatic tissues. Also, our results should not be extrapolated to all bivalvian species e.g., *Venerupis philipinarium*, where gonad is located within main body of the clams and signal from male mtDNA is predominant in most of male somatic tissues (Ghiselli, Milani & Passamonti, 2011).

### Question: Is this protein active?

In-gel detection of ATP synthase activity and two-dimensional Blue Native and SDS polyacrylamide electrophoreses suggested that proteins detected by anti-MATP8 and anti-FATP8 antibodies are integral parts of the ATPase complex in *M. edulis*. Straightforward blotting of Blue Native gels also supported these results.

## Conclusions

Based on protein sequence similarities (Fig. 1) and the results above, we consider it likely that active M and FATP8 proteins are present not only in *M. edulis* but through the genus *Mytilus*. Even though this gives us no right to claim that the whole Bivalvia class possesses an *atp8* gene, we strongly encourage scientists to focus more attention on the subject of presence and absence of this gene in bivalve mitochondrial genomes. Especially because similar *atp8* annotation problems have been observed in other organisms (flatworms (Nickisch-Rosenegk, Brown & Boore, 2000; Egger, Bachmann & Fromm, 2017) and nematodes (Hyman, 1988; Lavrov & Brown, 2001)) and because only proteomic based experimental approaches are capable of unambiguously resolving issues concerning “uncertain” protein genes.

##  Supplemental Information

10.7717/peerj.4897/supp-1 Data S1 Bivalvian mtDNA with and without anotteted *atp8* List of accession numbers.

10.7717/peerj.4897/supp-2 Supplemental Information 1 Part A of Figure 4

10.7717/peerj.4897/supp-3 Supplemental Information 2 Part B of Figure 4

10.7717/peerj.4897/supp-4 Supplemental Information 3 Part C of Figure 4

## Additional Information and Declarations

### Data Availability

The following information was supplied regarding data availability: Bivalvian Mitochondrial DNA sequences are available at GenBank with accession numbers: AB055624-NC_036488. See Supplemental Information 1 for a full list of accession numbers. The raw data are included in the Figures in the manuscript.
