# Supplementary figures and images for: Actively transcribed and expressed atp8 gene in Mytilus edulis mussels
Source: PeerJ. 2018 Jun 8;6:e4897. doi: 10.7717/peerj.4897 (PMC5995098; doi:10.7717/peerj.4897)

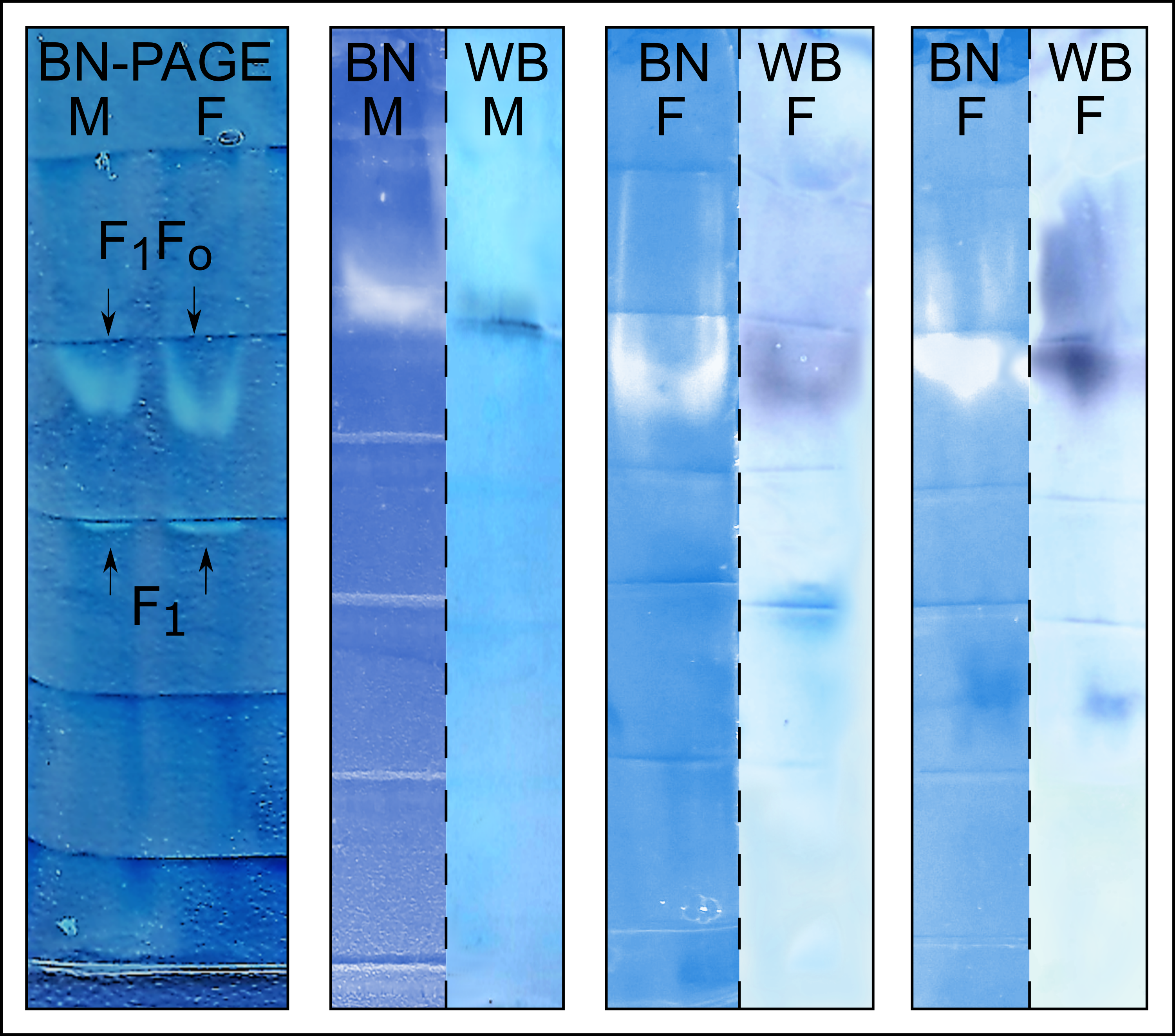

Supplement: Supplemental Information 1 [file peerj-06-4897-s002.png]

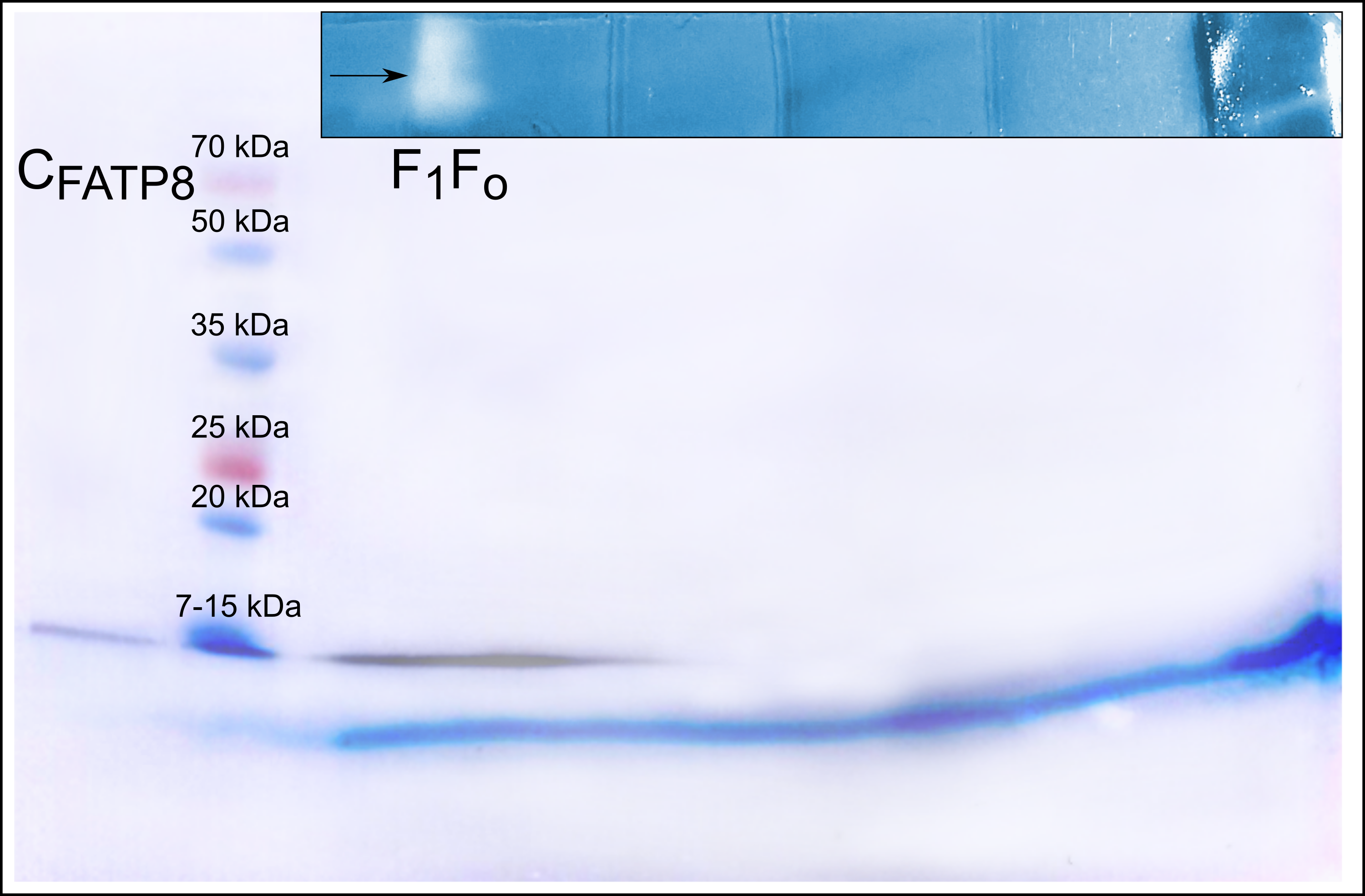

Supplement: Supplemental Information 2 [file peerj-06-4897-s003.png]

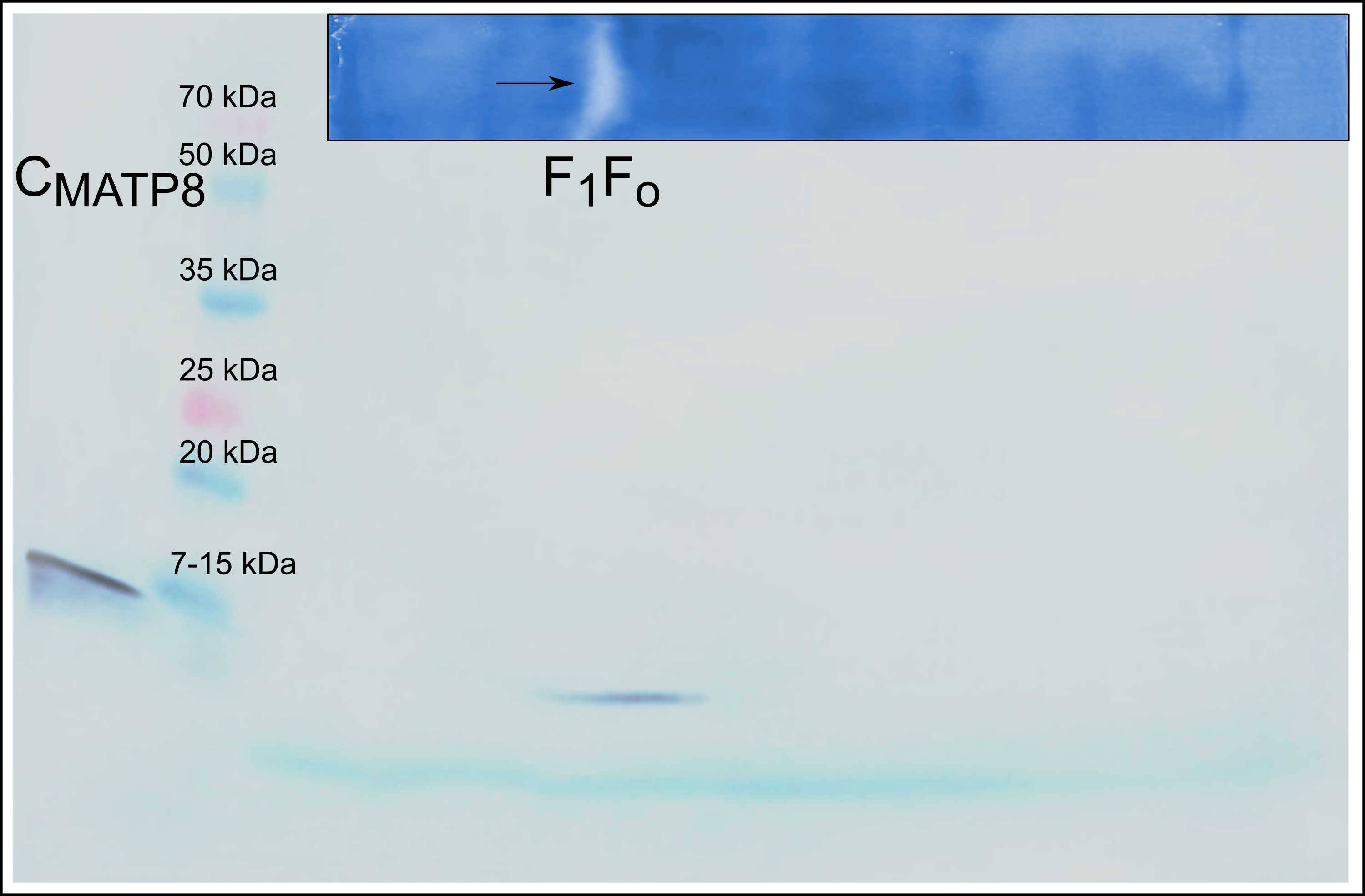

Supplement: Supplemental Information 3 [file peerj-06-4897-s004.png]
